# Faecal regenerating 1B protein concentration is not associated with child growth in rural Malawi

**DOI:** 10.1111/jpc.15231

**Published:** 2020-10-28

**Authors:** Zhifei Liu, Yue‐Mei Fan, Per Ashorn, Yin Bun Cheung, Lotta Hallamaa, Heikki Hyöty, Kenneth Maleta, Kirsi‐Maarit Lehto, Sami Oikarinen, Seppo Parkkila, Ulla Ashorn

**Affiliations:** ^1^ Center for Child Health Research, Faculty of Medicine and Health Technology Tampere University Tampere Finland; ^2^ Department of Paediatrics Tampere University Hospital Tampere Finland; ^3^ Program in Health Services and Systems Research and Center for Quantitative Medicine Duke‐NUS Medical School Singapore Singapore; ^4^ Fimlab Ltd Tampere University Hospital Tampere Finland; ^5^ Department of Public Health, School of Public Health & Family Medicine, College of Medicine University of Malawi Zomba Malawi

**Keywords:** child growth, intestinal repair, regenerating 1B protein, rural Malawi

## Abstract

**Aim:**

This study was designed to determine whether faecal regenerating 1B protein (REG1B) concentration is associated with physical growth among 6–30‐month‐old children in rural Malawi.

**Methods:**

This was a secondary analysis from a randomised controlled trial in rural Malawi in which we followed‐up 790 live‐born infants from birth to 30 months of age. We collected anthropometric data at the age of 6, 12, 18, 24 and 30 months. We measured faecal REG1B concentration by enzyme‐linked immunosorbent assay (ELISA) technique using stool samples collected at 6, 18 and 30 months of age. We assessed the association between faecal REG1B concentration and children's physical growth using linear regression and longitudinal data analysis.

**Results:**

Of 790 live‐born infants enrolled, 694 (87%) with at least one faecal REG1B concentration measurement were included in the analysis. Faecal REG1B concentration was not associated with the children's concurrent length‐for‐age z‐score (LAZ), weight‐for‐age z‐score (WAZ), weight‐for‐length z‐score (WLZ) and mid‐upper arm circumference‐for‐age z‐score (MUACZ) at any time point (*P* > 0.05), nor with a change in their anthropometric indices in the subsequent 6‐month period (*P* > 0.05).

**Conclusions:**

Faecal REG1B concentration is not associated with LAZ, WAZ, WLZ and MUACZ among 6–30‐month‐old infants and children in rural Malawi.

## What is already known on this topic

1. Linear growth faltering, i.e., stunting, commonly affects children in Sub‐Saharan Africa and other low‐resource settings.
2. Intestinal inflammation and environmental enteric dysfunction are believed to play an important role in contributing to childhood stunting.
3. Faecal concentration of regenerating 1B protein (REG1B), an intestinal repair marker, has been shown to predict stunting among infants and young children in Peru and Bangladesh.

## What this paper adds

1. Faecal REG1B concentration was not associated with attained size among 6‐, 18‐ or 30‐month‐old children in rural Malawi.
2. Faecal REG1B concentration was not associated with subsequent growth among 6–30‐month‐old children in rural Malawi.
3. Faecal REG1B concentration may not be a sensitive biomarker of child growth in rural Sub‐Saharan Africa.

Childhood stunting, defined as being too short for age, is estimated to affect globally 149 million children under 5 years old. Ninety per cent of these children live in Southern Asia and Sub‐Saharan Africa.^1^ Stunting can contribute to serious consequences, such as increased mortality, poor cognition, chronic diseases and even low career attainment in adults.^2^, ^3^, ^4^, ^5^, ^6^ Therefore, there is a pressing need to prevent childhood stunting.

One approach for prevention is identifying those children who are at the highest risk of stunting and targeting interventions to them. Measuring biomarkers from stool samples provides a non‐invasive way to assess the risk of growth faltering.^7^ Regenerating 1B protein (REG1B), related to intestinal repair, is one such possible biomarker. Its stool concentration has been shown to reflect intestinal inflammation which is a main contributor to stunting.^8^, ^9^, ^10^, ^11^ A cohort study in Bangladesh found that faecal REG1B concentration was associated with growth shortfall among children under 2 years old. Additionally, a similar association was found among children in Peru.^12^ However, there is still limited evidence on the association between REG1B and linear growth and none from Africa.

The aim of our analysis was to analyse REG1B concentration from stool samples and determine whether faecal REG1B concentration was associated with physical growth of infants and young children in rural Malawi. Specifically, we were interested in assessing the association of REG1B concentration with attained body size and change in body size.

## Materials and Methods

### Study site and design

This is a secondary analysis of data that were collected in a randomised controlled trial conducted in two hospitals (Mangochi, Malindi) and two health centres (Lungwena, Namwera) in Mangochi District, rural Malawi, South‐East Africa between February 2011 and April 2015. Details of this trial have been described elsewhere.^13^ The total population in the study area was about 190 000 and most of them spoke Chiyao and subsisted on farming and fishing.

In brief, pregnant women with less than 20 completed gestation weeks were enrolled and randomly allocated into three groups, receiving daily 60 mg iron +400 μg folic acid (IFA) in IFA group, a tablet of multiple micronutrients (MMN) in MMN group or 20 g of lipid‐based nutrient supplements (LNS) in LNS group as interventions. After delivery, 790 live‐born infants were followed up until age of 30 months. Clinic and home visits were conducted to collect both data using questionnaires and biological samples. Further details of trial design and its main outcomes have been published earlier.^14^ The study was approved by ethics committees in Malawi (College of Medicine) and Finland (Pirkanmaa Hospital District) and performed in accordance with the principles of Helsinki declaration and regulatory guidelines in Malawi. Written informed consents were obtained from caregivers.

### Stool samples collection

Research Assistants collected stool samples which had been placed in collection containers by mothers on the same day during home visits at 6, 18 and 30 months. The samples were on receipt immediately put in cooler bags. If the child had diarrhoea, sample collection was postponed by 2 weeks. The Research Assistants transported the samples in cooler bags to the site laboratory and laboratory technicians aliquoted the samples to cryovial tubes and stored them first in a −20°C freezer. Within 48 h, the samples were transported to a central laboratory where they were frozen at −80°C until being shipped on dry ice to Tampere University of Finland for analysis.

### Measurement of REG1B concentration

An enzyme‐linked immunosorbent assay (ELISA) technique (TECHLAB, Inc., Blacksburg, VA, USA) was used to quantify REG1B concentration in stool samples. Samples were diluted at 1:10 000 before adding 100 μL of standards, controls and stool samples in duplicates to plates with pre‐coated immobilised polyclonal antibody against REG1B. The plates were incubated at 37°C for 20 min followed by shaking and five times of washing before adding conjugate solution into each well. The incubation and washing were then repeated before adding substrate solution, followed by incubating for 15 min at room temperature. After adding stop solution, plates were read using optical density (OD) 450/620 nm (Multiskan FC Microplate Photometer, Thermo Fisher Scientific Inc., Waltham, MA, USA). The linear standard curve was made by plotting standard absorbance against standard concentration to calculate REG1B concentration. Concentration was expressed as μg/g.

### Anthropometric measurements

Anthropometric measurements of children were taken by trained study staff at clinic visits at 6, 12, 18, 24 and 30 months. The staff measured length or height to 1 mm using a length board (Harpenden Infantometer, Holtain Limited, Crosswell, UK) and weight with reading increments of 10 g using an electronic infant weighing scale (SECA 735) and a digital adult weighing scale (SECA 874). Also, they measured mid upper arm circumference (MUAC) and head circumference to 1 mm with the use of non‐stretchable plastic insertion tapes. We calculated length‐for‐age z‐score (LAZ), weight‐for‐age z‐score (WAZ), weight‐for‐length z‐score (WLZ), head circumference‐for‐age z‐score (HCZ) and mid‐upper arm circumference‐for‐age z‐score (MUACZ) using World Health Organisation Child Growth Standards.^15^ Change in z‐score was calculated by subtracting the anthropometric z‐score at the end of the interval of interest from that at the beginning of the interval.

### Other information

Baseline information of mothers and infants was obtained at both home and clinical visits. Maternal body mass index (BMI) and HIV infection was assessed at enrolment. Maternal malaria was diagnosed by the Rapid Diagnosis Test using Clearview Malaria Combo (British Biocell International Ltd., Dundee, UK). Research nurses recorded duration of pregnancy, infant sex and birthweight. Trained study staff collected breastfeeding information using questionnaires. Information on household food insecurity, expressed as household food insecurity access scores,^16^ was also collected to assess the situation of household food intake in the past month.

### Statistical analysis

Statistical analyses were performed with STATA version 15.0 (StataCorp, College Station, TX, USA). The definition of age for 6, 12, 18, 24 and 30 months was 20–32 weeks, 46–58 weeks, 72–84 weeks, 98–110 weeks and 124–136 weeks, respectively. Linear regression models were used to analyse the association between REG1B concentration at 6, 18 and 30 months and anthropometric data at the same time point respectively, and the association between REG1B concentration at 6 or 18 months and change in anthropometric z‐score in subsequent 6 months. Random effects model was used to estimate the association between repeated anthropometric indices at 6, 18 and 30 months and repeated faecal REG1B concentration also from the same multiple time points. Models were adjusted for child age, birthweight, breastfeeding after delivery (yes/no), maternal HIV infection (positive/negative), child sex, duration of pregnancy, maternal malaria (positive/negative) and household food insecurity access scores. These variables were selected as potential confounders in advance of the analysis, as recommended in a recent textbook on statistical analysis of child growth.^17^ To better understand the impact of possible confounders, we present as also unadjusted analyses as Supplementary tables. For the repeated measurement analysis, adjustment for child age was included also in the sensitivity analysis because of the strong association between it and the children's intestinal biomarker concentration. The numbers of participants included were different in different models because of missing values in REG1B data at different visit times.

## Results

**Fig 1:**
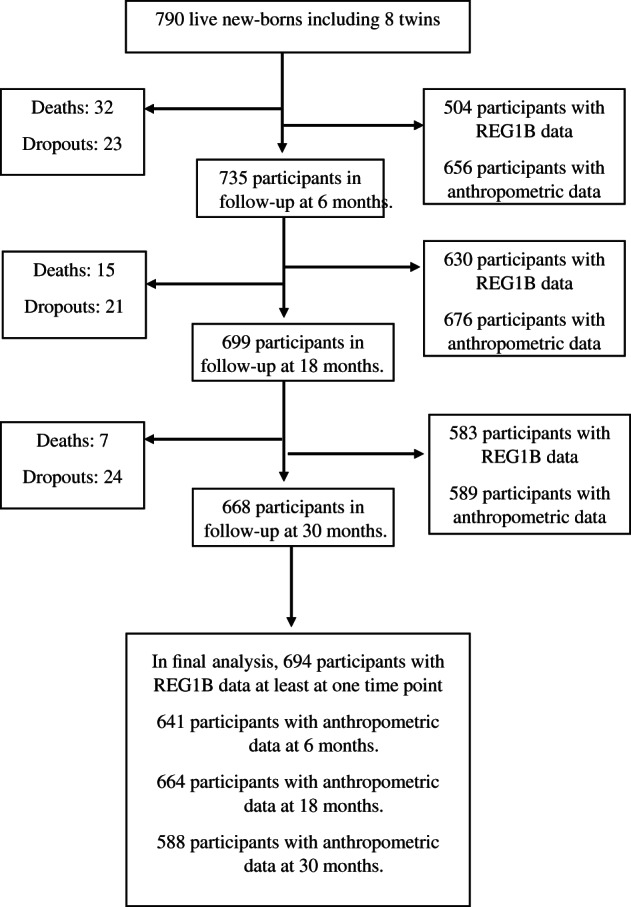
Participant flow.

Of the 790 live‐born infants enrolled in the study, 735 (93%) were still in follow‐up at 6 months, 699 (88%) at 18 months and 668 (85%) at 30 months of age. A total of 694 children (87% of those born alive) provided REG1B data on at least one time point and were thus included in the analysis (Fig. 1). Children whose data were not included in the analysis were born after a shorter mean duration of pregnancy, had smaller mean birthweight and were less frequently breastfed after birth than those included in the analysis (Table 1).

**Table 1:** Baseline characteristics of included and excluded participants†

Characteristics	Included (*n* = 694)	Excluded (*n* = 103)	*P*‐value‡
Infant and child characteristics			
Proportion of boys	47%	52%	0.575
Birthweight (kg)	2.97 (0.45)	2.69 (0.58)	<0.001
Proportion of breastfeeding after delivery	100%	88%	<0.001
Maternal characteristics			
Proportion with malaria parasitaemia	23%	24%	0.802
Proportion with low BMI (<18.5 kg m^−2^)§	8%	6%	0.554
Duration of pregnancy (weeks)	39.5 (1.8)	38.1 (3.9)	<0.001

†Values are mean (SD) or percentages. ‡ *P* value is obtained from Fisher's exact test for categorical variables, or Student's *t*‐test for continuous variables. §Body mass index (BMI) calculated by weight and height as kg m^−2^.

**Fig 2:**
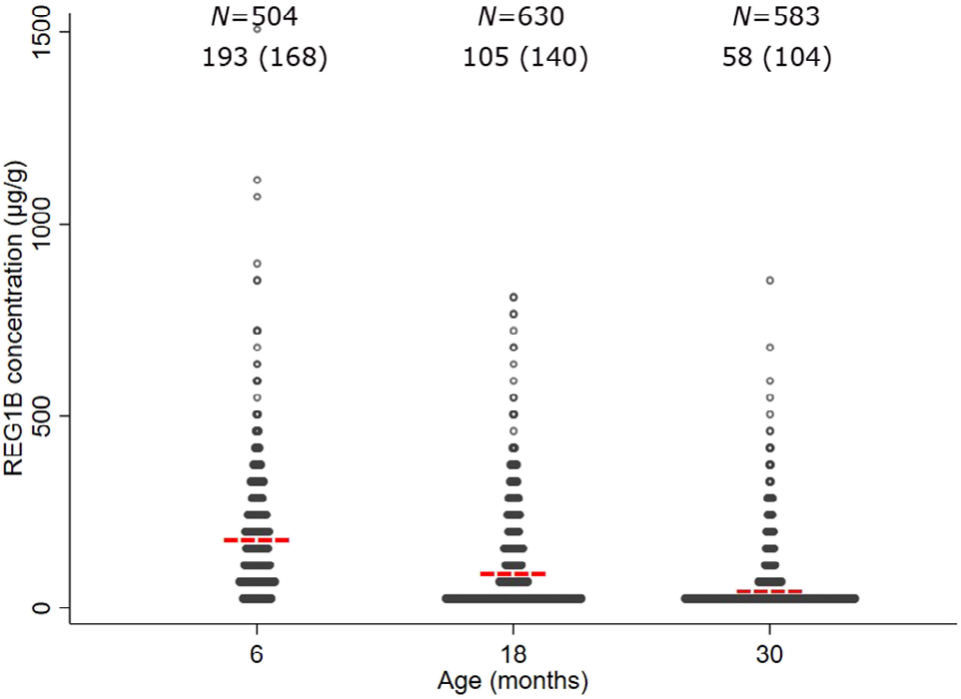
Distribution of faecal REG1B concentration at 6, 18 and 30 months. Mean REG1B concentration is shown as a red line for each age group. REG1B concentration ranges from 3.13 to 1530.07 μg/g. The values of mean (standard deviation) REG1B concentration at 6, 18 and 30 months are on the top of this figure. REG1B, regenerating 1B protein.

Mean (SD) REG1B concentration at 6, 18 and 30 months was 193 (168), 105 (140) and 58 (104) μg/g respectively (Fig. 2). Between 6 and 30 months, the mean LAZ decreased from −1.26 to −1.94, mean WAZ fell from −0.57 to −1.04 and mean HCZ decreased from −0.28 to −0.98. Mean MUACZ and mean WLZ were close to 0 throughout the follow‐up (Supplemental Table 1).

In cross‐sectional analyses, there were no statistically significant associations between faecal REG1B concentration and attained LAZ, WAZ, WLZ or MUACZ, at any age (*P* > 0.05; Table 2). For attained HCZ, there was a statistically significant, albeit weak, negative association at 6 months of age and a positive association at 30 months (*P* = 0.007 and 0.028, respectively; Table 2).

**Table 2:** The associations between children's faecal REG1B concentration and attained size at 6, 18 and 30 months of age†

Anthropometric index§	REG1B (every 100 μg/g)						
	6 months		18 months		30 months		
	B‡	95% CI	B‡	95% CI	B‡	95% CI	
LAZ	−0.00	−0.06, 0.05	0.03	−0.04, 0.09	0.04	−0.04, 0.11	
WAZ	−0.03	−0.09, 0.02	−0.02	−0.07, 0.04	0.04	−0.03, 0.11	
WLZ	−0.04	−0.10, 0.02	−0.04	−0.10, 0.01	0.02	−0.05 0.10	
HCZ	−0.08*	−0.13, −0.02	−0.00	−0.06, 0.05	0.08*	0.01, 0.16	
MUACZ	−0.06	−0.02, 0.01	−0.03	−0.08, 0.03	0.03	−0.04, 0.10	

*P < 0.05. †Models were adjusted for birthweight, breastfeeding after delivery (yes/no), maternal BMI, child sex, duration of pregnancy, maternal malaria status (positive/negative), maternal HIV status (positive/negative) and household food insecurity access scores. ‡Unstandardized regression coefficient between the children's faecal REG1B concentration at the age indicated in the column heading and their anthropometric index indicated in the left column. §HCZ, head circumference‐for‐age z‐score; LAZ, length‐for‐age z‐score; MUACZ, mid‐upper arm circumference‐for‐age z‐score; REG1B, regenerating 1B protein; WAZ, weight‐for‐age z‐ score; WLZ, weight‐for‐length z‐score.

There were no statistically significant associations between the participants' faecal REG1B concentration at 6 or 18 months and change in their LAZ, WAZ, WLZ, HCZ or MUACZ in the subsequent 6‐month period (Table 3).

**Table 3:** The associations between faecal REG1B concentration at 6‐ or 18‐month‐old children and their change in anthropometric z‐scores in the subsequent 6 months†

Change in anthropometric index§	REG1B (every 100 μg/g)			
	6 months		18 months	
	B‡	95% CI	B‡	95% CI
ΔLAZ	0.00	−0.04, 0.05	0.00	−0.03, 0.04
ΔWAZ	−0.00	−0.04, 0.03	0.01	−0.03, 0.04
ΔWLZ	−0.01	−0.05, 0.04	0.01	−0.04, 0.06
ΔHCZ	0.01	−0.02, 0.04	−0.01	−0.04, 0.02
ΔMUACZ	0.00	−0.05, 0.05	−0.00	−0.05, 0.04

†Models were adjusted for birthweight, breastfeeding after delivery (yes/no), maternal BMI, child sex, duration of pregnancy, maternal malaria status (positive/negative), maternal HIV status (positive/negative) and household food insecurity access scores. ‡Unstandardized regression coefficient between the children's faecal REG1B concentration at the age indicated in the column heading and their gain in anthropometric index indicated in the left column. §ΔHCZ, change in head circumference‐for‐age z‐score; ΔLAZ, change in length‐for‐age z‐score; ΔMUACZ, change in mid‐upper arm circumference‐for‐age z‐score; REG1B, regenerating 1B protein; ΔWAZ, change in weight‐for‐age z‐score; ΔWLZ, change in weight‐for‐length z‐score.

In repeated measurements analysis, there were also no statistically significant associations between faecal REG1B and LAZ, WAZ, WLZ, MUACZ or HCZ after adjusting for age, birthweight, breastfeeding, maternal BMI, child sex, duration of pregnancy, maternal malaria status, maternal HIV status and household food insecurity access scores (Table 4).

**Table 4:** The association between repeated faecal REG1B concentration and repeated anthropometric z‐scores at 6, 18 and 30 months†

Anthropometric index§	REG1B (every 100 μg/g)	
	Coefficient (SE)‡	95% CI
LAZ	0.01 (0.01)	−0.01, 0.03
WAZ	−0.01 (0.01)	−0.04, 0.01
WLZ	−0.02 (0.02)	−0.06, 0.01
HCZ	−0.01 (0.01)	−0.03, 0.01
MUACZ	−0.02 (0.01)	−0.05, 0.01

†Random effects models were adjusted for age, birthweight, breastfeeding after delivery (yes/no), maternal BMI, child sex, duration of pregnancy, maternal malaria status (positive/negative), maternal HIV status (positive/negative) and household food insecurity access scores. ‡The coefficient of the association between the children's faecal REG1B concentration and anthropometrics from random effects models, SE, standard error. §HCZ, head circumference‐for‐age z‐score; LAZ, length‐for‐age z‐score; MUACZ, mid‐upper arm circumference‐for‐age z‐score; REG1B, regenerating 1B protein; WAZ, weight‐for‐age z‐score; WLZ, weight‐for‐length z‐score.

Unadjusted cross‐sectional analyses gave essentially similar results to the fully adjusted analyses (Supplemental Tables 2,3). Repeated measurement analyses that were adjusted only for the child age gave also other similar results to the fully adjusted model, except for MUACZ, for which there was a weak negative association with faecal REG1B concentration (Supplemental Table 4).

## Discussion

Our study aimed to analyse the association between faecal REG1B concentration and child growth in rural Malawian infants and children. In a sample of 694 children followed at 6, 18 and 30 months, faecal REG1B concentration was not associated with the children attained size at the same visit. Furthermore, faecal REG1B concentration at 6 or 18 months of age was not predictive of the children's growth in the subsequent 6 months. Finally, a repeated measurements analysis showed only few associations between the participants' faecal REG1B concentration and their anthropometric measurements. For attained HCZ, there was a weak negative association at 6 months of age and a positive association at 30 months, suggesting a spurious finding. Thus, our sample findings suggest that faecal REG1B concentration does not function well as a biomarker that would be predictive of infant or young child growth in rural Malawi.

In our study, the results were obtained from a longitudinal study design with a large sample size. In principle, problems related to stool sample collection, storage or analysis could introduce a bias for interpretation of the findings. However, the samples were collected only from children with no diarrhoea, aliquoted after collection and stored at −80°C, at which temperature biological specimens can be maintained over many years.^18^ For REG1B concentration analysis, we used ELISA technique that has been proven a reliable method to detect protein in stool samples.^12^, ^19^ Furthermore, we did the analysis in duplicate to reduce potential variation during laboratory work. Even though some cohort members were not included in this analysis, the variables that showed a difference between the included and excluded were adjusted for in the analytic models. Therefore, we consider the sample findings unbiased and indicative of a lack of association between faecal REG1B concentration and physical growth in the Malawian population.

Our findings from Malawi contrast those from two previous studies in Bangladesh and Peru, both using the same REG1B ELISA test as we used. In those studies, faecal REG1B concentration in early infancy was predictive of growth faltering by 2 years of age.^12^ Increased faecal REG1B concentration has also been associated with other conditions that predict low LAZ, such as small intestine bacterial overgrowth among 2‐year‐old Bangladeshi children^19^ and presence of IgA class antibodies against lipopolysaccharide in the plasma of 6–10‐month‐old Pakistani infants.^20^ Besides different study locations between our and the earlier studies, there were differences in the mean faecal REG1B concentration (higher in Malawi than elsewhere), the age when REG1B concentration was measured and the duration of the participants' follow‐up. There may also have been additional differences in stool processing and storage, the participants' diets and background inflammation or other factors that may affect the REG1B protein or the assay. Given the limitations in data comparability, we cannot conclusively explain the observed differences. However, it seems that faecal REG1B concentration may not be a universally useful biomarker to predict linear growth in all infant and child populations.

REG1B is one of several potential biomarkers for intestinal inflammation and dysfunction. The other commonly reported biomarkers include calprotectin, neopterin, alpha‐1‐antitrypsin and myeloperoxidase.^21^ Whereas REG1B concentration is believed to indicate intestinal repair and regeneration, calprotectin, neopterin and myeloperoxidase measure mostly intestinal inflammation and alpha‐1‐antitrypsin reflects increased intestinal permeability.^22^ Each of these biomarkers can be reliably measured from fresh or stored stool samples, with a simple ELISA‐test. All except REG1B test are commercially available, with an approximate material cost of 5 USD per test. However, faecal calprotectin does not necessarily indicate intestinal inflammation correctly among under‐4‐year‐old children,^23^ and alpha‐1‐antitrypsin concentration may be affected by breastfeeding.^24^ There is also some evidence that faecal myeloperoxidase and neopterin analyses are difficult to interpret unless both tests are done concomitantly.^25^ Since REG1B is not known to have these limitations and its concentration has predicted infant growth in Bangladesh and Peru,^12^ it might still have a place in the assessment of intestinal health, especially among young breastfeeding infants.

In conclusion, our findings suggest that faecal REG1B concentration may be not a sensitive predictor of child growth in rural Malawi. However, given that there are no other studies published from Sub‐Saharan Africa, these data should be treated as preliminary. Further studies in other paediatric populations in Africa would be needed to establish the value of faecal REG1B concentration as a predictive biomarker for child growth in Sub‐Saharan Africa.

## Supporting information


**Supplemental Table 1** Mean (SD) anthropometric z‐scores among study participants at 6, 18 and 30 months of age ^†^
**Supplemental Table 2**. Unadjusted association between children's faecal REG1B concentration and attained size at 6, 18 and 30 months of age ^†^
**Supplemental Table 3**. Unadjusted associations between faecal REG1B concentration at 6‐ or 18‐month‐old children and their change in anthropometric z‐scores in the subsequent 6 months ^†^
**Supplemental Table 4**. The association between repeated faecal REG1B concentration and repeated anthropometric z‐scores at 6, 18 and 30 months^†^
