# Supplementary material for: Faecal regenerating 1B protein concentration is not associated with child growth in rural Malawi
Source: J Paediatr Child Health. 2020 Oct 28;57(3):388–94. doi: 10.1111/jpc.15231 (PMC8048694; doi:10.1111/jpc.15231)
Supplement: Supplementary file 1 — Supplemental Table 1 Mean (SD) anthropometric z‐scores among study participants at 6, 18 and 30 months of age † Supplemental Table 2. Unadjusted association between children's faecal REG1B concentration and attained size at 6, 18 and 30 months of age † Supplemental Table 3. Unadjusted associations between faecal REG1B concentration at 6‐ or 18‐month‐old children and their change in anthropometric z‐scores in the subsequent 6 months † Supplemental Table 4. The association between repeated faecal REG1B concentration and repeated anthropometric z‐scores at 6, 18 and 30 months† [file JPC-57-388-s001.docx]

## Supplemental Table 1. Mean (SD) anthropometric z-scores among study participants at 6, 18 and 30 months of age ^†^

| Anthropometric  Index ^‡^ | Child age | | |
| --- | --- | --- | --- |
|  | 6 months | 18 months | 30 months |
| LAZ | -1.26 (1.14) | -1.66 (1.09) | -1.94 (0.95) |
| WAZ | -0.57 (1.19) | -0.90 (1.03) | -1.04 (0.94) |
| WLZ | 0.38 (1.15) | -0.15 (0.99) | 0.04 (0.94) |
| HCZ | -0.28 (1.06) | -0.82 (0.99) | -0.98 (0.95) |
| MUACZ | 0.06 (1.18) | 0.06 (0.99) | -0.09 (0.92) |

^†^ Values are mean (SD).

^‡^ HCZ, head circumference-for-age z-score; LAZ, length-for-age z-score; MUACZ, mid-upper arm circumference-for-age z-score; SD, standard deviation; WAZ, weight-for-age z score; WLZ, weight-for-length-age z-score.

## Supplemental Table 2. Unadjusted association between children’s fecal REG1B concentration and attained size at 6, 18 and 30 months of age ^†^

| Anthropometric  Index ^§^ | REG1B (every 100 µg/g) | | | | | | |
| --- | --- | --- | --- | --- | --- | --- | --- |
|  | 6 months | | 18 months | | 30 months | | |
|  | B ^‡^ | 95% CI | B ^‡^ | 95% CI | B ^‡^ | 95% CI |  |
| LAZ | 0.00 | -0.06, 0.06 | 0.02 | -0.05, 0.08 | 0.01 | -0.07, 0.08 | |
| WAZ | -0.04 | -0.10, 0.03 | -0.03 | -0.09, 0.03 | 0.04 | -0.06, 0.09 | |
| WLZ | -0.05 | -0.11, 0.01 | -0.05 | -0.11, 0.00 | 0.01 | -0.06 0.09 | |
| HCZ | -0.05 | -0.11, 0.00 | 0.00 | -0.05, 0.06 | 0.07 | -0.01, 0.14 | |
| MUACZ | -0.06 | -0.12, 0.00 | -0.04 | -0.10, 0.02 | 0.02 | -0.05, 0.10 | |

^†^ Associations were assessed using unadjusted linear regression.

^‡^ Unstandardized regression coefficient between the children’s fecal REG1B concentration at the age indicated in the column heading and their anthropometric index indicated in the left column.

^§^ HCZ, head circumference-for-age z-score; LAZ, length-for-age z-score; MUACZ, mid-upper arm circumference-for-age z-score; REG1B, regenerating 1B protein; WAZ, weight-for-age z- score; WLZ, weight-for-length z-score.

## Supplemental Table 3. Unadjusted associations between fecal REG1B concentration at 6- or 18-month-old children and their change in anthropometric z-scores in the subsequent 6 months ^†^

| Change in anthropometric  Index ^§^ | REG1B (every 100 µg/g) | | | |
| --- | --- | --- | --- | --- |
|  | 6 months | | 18 months | |
|  | B ^‡^ | 95% CI | B ^‡^ | 95% CI |
| ΔLAZ | 0.01 | -0.03, 0.05 | -0.01 | -0.04, 0.03 |
| ΔWAZ | 0.00 | -0.03,0.04 | 0.01 | -0.03, 0.04 |
| ΔWLZ | -0.00 | -0.04, 0.04 | 0.01 | -0.03, 0.06 |
| ΔHCZ | 0.00 | -0.03, 0.04 | -0.01 | -0.03, 0.02 |
| ΔMUACZ | 0.01 | -0.04, 0.05 | 0.00 | -0.04, 0.05 |

^†^Associations were assessed using unadjusted linear regression models.

^‡^ Unstandardized regression coefficient between the children’s fecal REG1B concentration at the age indicated in the column heading and their gain in anthropometric index indicated in the left column.

^§^ ΔHCZ, change in head circumference-for-age z-score; ΔLAZ, change in length-for-age z-score; ΔMUACZ, change in mid-upper arm circumference-for-age z-score; REG1B, regenerating 1B protein; ΔWAZ, change in weight-for-age z-score; ΔWLZ, change in weight-for-length z-score.

## Supplemental Table 4. The association between repeated fecal REG1B concentration and repeated anthropometric z-scores at 6, 18 and 30 months ^†^

| Anthropometric index ^§^ | REG1B (every 100 µg/g) | |
| --- | --- | --- |
|  | Coefficient (SE) ^‡^ | 95% CI |
| LAZ | 0.01 (0.01) | -0.01, 0.03 |
| WAZ | -0.02 (0.01) | -0.04, .0.01 |
| WLZ | -0.02 (0.02) | -0.05, 0.01 |
| HCZ | -0.01 (0.01) | -0.02, 0.01 |
| MUACZ | -0.04 (0.02) * | -0.07, -0.01 |

^†^ Random effects models were adjusted for age.

^‡^ The coefficient of the association between the children’s fecal REG1B concentration and anthropometrics from random effects models, SE, standard error.

^§^ HCZ, head circumference-for-age z-score; LAZ, length-for-age z-score; MUACZ, mid-upper arm circumference-for-age z-score; REG1B, regenerating 1B protein; WAZ, weight-for-age z-score; WLZ, weight-for-length z-score.

**P*<0.05.
